# Corneal topographic changes in premenopausal and postmenopausal women

**DOI:** 10.1186/1471-2415-7-9

**Published:** 2007-05-14

**Authors:** Erdinc Aydin, Helin Deniz Demir, Fazli Demirturk, Ahmet Cantug Calıskan, Hakan Aytan, Unal Erkorkmaz

**Affiliations:** 1 Department of Ophthalmology, Gaziosmanpasa University Faculty of Medicine, Tokat, Turkey; 2 Department of Obstetrics and Gynecology, Gaziosmanpasa University Faculty of Medicine, Tokat, Turkey; 3 Department of Biostatistics, Gaziosmanpasa University Faculty of Medicine, Tokat, Turkey

## Abstract

**Background:**

To asses the effect of menopause on the corneal curvature changes using corneal computerized videokeratography (CVK) in premenopausal and postmenopausal healthy women.

**Methods:**

Thirty-six postmenopausal women with mean ages of 49.2 (range 39 to 57) were enrolled in this randomized, prospective study, comparing with 26 healthy controls with mean ages of 38.5 +/- 4.9 (range 32 to 49). Subjects were determined to be postmenopausal, by the Gynecology and Obstetrics Department, based on blood Follicular Stimulating Hormone (FSH), Luteinizing Hormone (LH), Estradiol, Progesterone levels and clinical complaints. Complete ophthalmic examination and CVK using Haag-Streit System was performed in both premenopausal and postmenopausal women.

**Results:**

Mean horizontal curvature and vertical curvature of central corneal power in premenopausal women were 43.5 +/- 1.25 Diopter (D), and 44.1 +/- 1.53 D. Mean horizontal curvature and vertical curvature of central corneal power in postmenopausal women were 43.9 +/- 1.4 D, and 44.6 +/- 1.3 D. The mean keratometric astigmatisms of premenopausal and postmenopausal women were 0.81 +/- 0.57 D (4–179 degrees), 0.74 degrees +/- 0.5 D (1–180 degrees) respectively. No significant corneal curvature changes were detected between premenopausal and postmenopausal groups (P > 0.05). On the other hand, we only found negative but significant correlation between horizontal corneal curvature and estrogen level of postmenopausal women (r = -0.346, p = 0.038).

**Conclusion:**

Menopause is physiologic process and may also affect corneal topographic changes. In postmenopausal women, corneal steeping was observed minimally compared to premenopausal women. The results suggest that changes in estrogen level of women with menopause are associated with slightly alteration of horizontal curvature of cornea.

## Background

Corneal functions can be regulated by sex hormones that have been reported by epidemiologic studies; however, the mechanism of this phenomenon is not apparent. The estrogen receptor (ER), progesterone receptor (PR) and androgen receptor (AR) were found in the nuclei of human corneal epithelial, stromal and endothelial cells [1-3].

The corneal variables, which change during the menstrual cycle, include corneal thickness, curvature, and sensitivity. A change in thickness during the menstrual cycle of women has been reported; the percentage of increase in thickness was 5.6 % on days of 15 and 16 [4]. There are also reports that show an increase in the corneal thickness during pregnancy. An increase of 3 percent in corneal thickness with insignificant fluctuation through each trimester of pregnancy has been reported [5].

Kiely et al [6] detected steepening of central curvatures in both horizontal and vertical meridians at the beginning of the cycle and flattening of them after ovulation in healthy women. However until recent years, no report has been published about the changes of corneal curvature in postmenopausal women.

In the current study, we investigated the effect of menopause on corneal curvature changes, the relation between corneal topographic changes and sex-hormone levels in premenopausal and postmenopausal healthy women.

## Methods

Study design followed Institutional Research Ethics Board guidelines, and informed consent was obtained. This randomized, prospective study included twenty-six left eyes of 26 premenopausal and thirty-six left eyes of 36 postmenopausal healthy women were recruited for analysis of corneal curvature changes depending on sex hormones. Mean ages of premenopausal and postmenopausal subjects were 38.5 (range 32 to 49 years) and 49.2 (range 39 to 57 years). We excluded the patients with contact lenses, prior ocular surgery, dry eyes, inflammatory ocular disease, diabetes mellitus and hormone replacement therapy.

All subjects underwent the following routine examination by the same ophthalmologist and a certified ophthalmic technician, however the evaluation of their corneal topographies were done by different ophthalmologist as a blind study: (1) demographic and ocular history, (2) slit-lamp biomicroscopy (Topcon) (3) manifest refraction (fogging technique), (4) keratometry (Topcon) (5) videokeratography (CTK 922 Haag-Streit Ag, Liebefeld, Switzerland). In this system applanation tonometry was not performed prior to keratography. No artificial method for widening palpebral fissure (such as an eyelid speculum) was applied. All measurements were obtained with Haag-Streit corneal topographer. In this system the corneal curvature is divided into 30 concentric circles. The curvature is measured in dioptres (D), with each point representing 1 dioptre value, for a total of 7680 values (30*256). The numeric data of the topographic maps were computerized by a mathematical algorithm. One individual, specifically trained in the use of the Haag-Streit instrument, performed the topographical examinations. Testing was done until acceptable quality imaging was obtained. The values of quantitative topographic parameters were recorded as central power (CP) of the cornea along the optical axis of the instrument, expressed in dioptres. Hormone levels (Follicular Stimulating Hormone (FSH), Luteinizing Hormone (LH), Estradiol, and Progesterone) were determined after 8 hours of fasting. All hormone levels and CVK were analyzed at luteal phase (after ovulation) in premenopausal women. None of the subjects were prescribed to use hormone replacement therapy.

For statistical evaluation, the two independent sample t-tests were applied. Pearson correlation coefficient was performed for relation among cornea vertical curvature, cornea horizontal curvature and estrogen, progesterone hormone levels. Since there was significant difference between the ages of premenopausal and postmenopausal groups, other variabilities adjusted with age were evaluated by a multivariate analyze method (Hotelling T^2 ^test). All data were analyzed using Statistical Package program (SPSS inc. Chicago, IL.)

## Results

All subjects who met the criteria were consecutively recruited for the study from June 2005 to April 2006. Although there was no difference in the best-corrected visual acuity (BCVA) (P > 0.05), statistically significant difference was observed between the ages of premenopausal and postmenopausal groups (P <0.05) (Table 1).

**Table 1:** Demographic and refractive data for premenopausal and postmenopausal women

	**Premenopausal **(n = 26) (Mean ± SD)	**Postmenopausal **(n = 36) (Mean ± SD)	**P-value**
Age (year)	38.5 ± 4.9	49.2 ± 4.9	<0.001
BCVA (20/20 count)	26	36	1
Astigmatism	0.81 ± 0.57 D (4°–179°)	0.74 ± 0.5 D (1°–180°)	0.872
Horizontal curvature	43.5 ± 1.25 D	43.9 ± 1.4D	0.343 0.250*
Vertical curvature	44.1 ± 1.53D	44.6 ± 1.3D	0.170 0.352*
Estradiol level (pg/ml)	93.7 ± 33.4	22.37 ± 10.76	<0.001
Progesterone level (ng/ml)	6.36 ± 3.12	0.24 ± 0.11	<0.001
Fasting blood Sugar (mg/dl)	97.20 ± 7.46	100.86 ± 7.36	0.059 0.065*

*: Adjusted with age (Multivariate Analysis result)

SD: Standard deviation, BCVA: Best corrected visual acuity (Snellen), D: Diopter

Mean horizontal curvature and vertical curvature of central corneal power in premenopausal women were 43.5 ± 1.25 D, 44.1 ± 1.53D respectively. Mean horizontal curvature and vertical curvature of central corneal power in postmenopausal women were 43.9 ± 1.4D, 44.6 ± 1.3D; the mean keratometric astigmatisms of premenopausal and postmenopausal women were 0.81 ± 0.57D (4°–179°), 0.74 ± 0.5D (1°–180°) respectively (Table 1).

**Figure 1:**
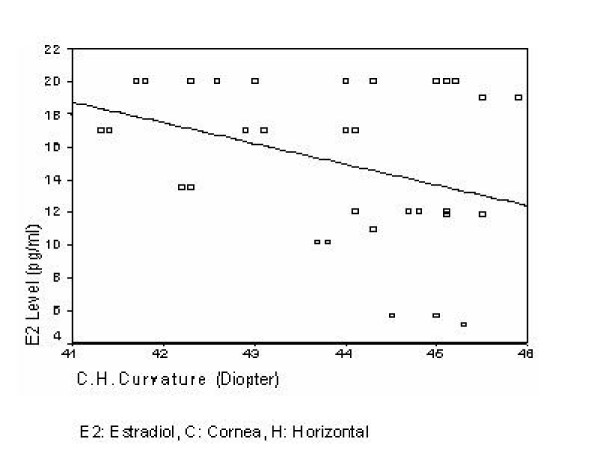
The correlation between estradiol and cornea horizontal curvature in postmenopausal women.

We did not find significant corneal curvature changes between premenopausal and postmenopausal women (P > 0.05). On the other hand, when we investigated any correlation between sex-hormones and corneal changes, we detected negative but significant correlation between horizontal corneal curvature and estrogen levels of postmenopausal women (r = -0.346, p = 0.038) (Figure 1). There was no correlation among vertical, horizontal curvature and progesterone level of premenopausal and postmenopausal women.

The data on vertical, horizontal curvature of cornea and and fasting blood sugar levels has been included in multivariate analyze and there was no significant difference in vertical, horizontal curvature and fasting blood glucose levels adjusted with age between premenopausal and postmenopausal groups (P = 0.352, P = 0.250, P = 0.065, respectively).

## Discussion and conclusion

Estrogen receptors, ARs and PRs have been detected in the human lacrimal [7,8] and meibomian glands [9] and have been implicated in dry eye [10,11]. They were also found in the nuclei of human corneal epithelial, stromal and endothelial cells [1-3].

El Hage and Beaulne [12] reported that significant changes in corneal topography occurred in relation with time during menstrual cycle, with greatest alterations occurring in the peripheral cornea. However, no significant changes with time were detected for central and peripheral corneal thickness measurement of eight women. Kiely at al [6] investigated significant corneal steeping at the beginning of the cycle in six women, in addition cyclic changes in corneal thickness during the menstrual cycle, and thickening of the cornea at ovulation. Soni et al [13] found that female corneas attained minimal thickness just before ovulation and maximal thickness at the beginning or end of the menstrual cycle. During pregnancy, Weinreb et al [14] detected an increase in corneal thickness. Indeed, these reports seem to show a strong association between corneal thickness and female hormone levels, particularly estrogen level. In this circumstance, it is not apparent whether hormonal influence is exerted through direct interaction in the cornea, or via secondary effects such as systemic water retention by estrogen-induced up regulation of the rennin-aldosterone system [15]. Moreover, we have not found any published research in literature related with the effect of menopause on any changes of corneal curvature.

Corneal Topography provides a detailed depiction of the corneal surface displaying its curvature, dioptres and radial directions [16]. Steepening of central curvatures in both horizontal and vertical meridians at the beginning of the menstrual cycle, flattening of curvature after ovulation in both meridians of healthy women was reported [6]. In the present study, though no significant correlations were found among estrogen, progesterone and corneal curvatures in premenopausal women, we investigated negative but significant correlation between estrogen levels and horizontal corneal curvatures in postmenopausal women. In the sight of these findings, we speculate that decrease in estrogen level of women in postmenopausal term may cause steeping of corneal horizontal curvature.

Previous studies reported that astigmatism changes from with the rule astigmatism, which is dominant in younger ages, to against the rule astigmatism with aging [17,18]. Saunders implied that a gradual change of the astigmatic axis reached an oblique mid-point in early middle life and presented a new regression equation for the change of astigmatism [19]. Hayashi et al evaluated the aging changes in corneal shape and detected that the averaged maps of subjects from < 20 years of age to the 40 s showed a vertical bow-tie-rule astigmatism. In the maps of subjects in their 50 s and 60 s, the central steep area gradually extended horizontally until it became a round configuration [20]. In our study, we did not find significant astigmatic shift within premenopausal and postmenopausal groups, but detected with-the-rule astigmatism in young subject and oblique or against-the-rule astigmatism older subjects. Mean astigmatism values also were similar in both groups.

We considered that the present study was performed as one masked, preliminary on limited number of subjects, might result in biases; therefore more clinical trials are needed with larger series of premenopausal, postmenopausal groups and further follow-up time from premenopausal to postmenopausal term.

## Competing interests

The author(s) declare that they have no competing interests.

## Authors' contributions

**EA **Conceived the study, participated in its design, data acquisition, data analysis, literature search, main paper writing and submission.

**HDD **Participated in its design, literature search and paper modifications.

**FD, ACC, and HA **Data acquisition.

**UE **Performed all the statistical analysis.

## Pre-publication history

The pre-publication history for this paper can be accessed here:


